# Physician’s Perceptions of Telemedicine in HIV Care Provision: A Cross-Sectional Web-Based Survey

**DOI:** 10.2196/publichealth.6896

**Published:** 2017-05-30

**Authors:** Kelly Anderson, Troy Francis, Francisco Ibanez-Carrasco, Jason Globerman

**Affiliations:** ^1^ St. Michael's Hospital Family Health Team Department of Family and Community Medicine Toronto, ON Canada; ^2^ Leslie Dan Faculty of Pharmacy University of Toronto Toronto, ON Canada; ^3^ Ontario HIV Treatment Network Toronto, ON Canada

**Keywords:** HIV, AIDS, telemedicine, health surveys

## Abstract

**Background:**

Telemedicine, or electronic interactive health care consultation, offers a variety of benefits to both patients and primary care clinicians. However, little is known about the opinions of physicians using these modalities.

**Objective:**

The aim of this study was to examine physician perceptions, including challenges, risks, and benefits of the use of telemedicine in human immunodeficiency virus (HIV) patient care.

**Methods:**

A Web-based, self-administered, anonymous, cross-sectional survey was sent to physicians known to be providing medical care to patients living with HIV in Ontario, Canada. Descriptive statistics and frequencies were used to examine physician perceptions and characteristics of participants.

**Results:**

Among the 51 invited participants, 48 (94%) completed the survey. Sixty-two percent (29/47) of respondents reported that they used some form of telemedicine to care for HIV patients in their practice. Of the respondents who identified as having used telemedicine in their practice, telephone (86%, 25/29), email (69%, 20/29), and teleconsultation (24%, 7/29) were listed as frequent modalities used. A significant number of physicians (83%, 38/46) agreed that an obstacle to adopting telemedicine is their perception that this modality does not allow for a comprehensive assessment of their patients’ health. In addition, 65% (28/43) of physicians agreed that patients may not feel adequately connected to them as a provider if they used telemedicine. However, 85% (39/46) of respondents believed that telemedicine could improve access and timeliness to care along with increasing the number of times physicians can interact with their patients.

**Conclusions:**

From the perceptions of physicians, telemedicine shows promise in the care of patients living with HIV. More than half of the respondents are already using telemedicine modalities. Whereas many physicians are concerned about their ability to fully assess the health of a patient via telemedicine, most physicians do see a need for it—to reduce patient travel times, reduce exposure to stigma, and improve efficiency and timely access to care. Challenges and risks such as technological gaps, confidentiality, and medicolegal concerns must be addressed for physicians to feel more comfortable using telemedicine.

## Introduction

Telemedicine, or electronic interactive health care consultation, offers a variety of benefits to both patients and primary care clinicians [1]. Telemedicine encompasses a wide variety of health care services, and with the current advances in technology, these services are quickly evolving and becoming more affordable and accessible. Telemedicine models are also wide ranging: from live synchronous connections between two or more parties in a health encounter to asynchronous training modules; from programs delivered with a desktop in one place to just-in-time learning delivered via mobile devices [2-4]. In all of these cases, there are a variety of potential obstacles and challenges such as technical (eg, type of device and data plans), administrative (coordination and support), financial (costs of technology), and cultural (affinity with some media over other) [5]. The complexity of the illness is a barrier that also needs to be addressed when identifying which telemedicine model is most appropriate to use. Although telemedicine is promising in the provision of services to patients, the evidence remains limited and inconsistent [5].

Human immunodeficiency virus (HIV) is a highly complex chronic illness and should be thought of as a myriad of conditions and not one single issue. Many patients suffering from HIV are also afflicted with physical and psychological impairments, making the use of telemedicine in this population a strong compliment to current treatment options [6,7]. Although various telemedicine modalities are well received by patients—including people living with HIV—due to its convenience, ability to increase confidentiality, and reduce stigma, little is known about what physicians providing HIV care think about telemedicine [8-11]. The uptake of telemedicine services relies on system structures being embedded within medical practice and physicians playing a pivotal role in adopting the technology [12,13]. Physicians’ acceptance of telemedicine is essential in its use, and thus understanding physicians’ perceptions on the use of, and the skills required to use telemedicine to care for those living with HIV, may help identify the perceived challenges, risks, and benefits to the uptake of its many modalities. Additionally, identifying physician characteristics may shed light into how often HIV care providers use telemedicine and what impact it has on their practice and on the health of patients. Accordingly, the purpose of this study was to explore how physicians perceive the use of telemedicine in HIV care in the province of Ontario. This is a necessary entry point into understanding whether HIV disease itself can and must be treated using telemedicine or specialized physicians in general, who use telemedicine, need to be trained in the complexity of HIV to better serve those they see who happen to be HIV positive as well.

## Methods

### Participants

Physicians who were providing medical care to people living with HIV in Ontario, Canada were asked to complete a Web-based, self-administered, and anonymous survey regarding the use of telemedicine in HIV care.

### Recruitment

A list of all registered infectious disease specialists in the province (n=218) was obtained via the College of Physicians and Surgeons of Ontario. Of these, 51 individuals were identified as providing HIV-specific care. A link to the survey was emailed to these individuals. In addition, 2 additional email reminders were circulated 1 week apart to get as many responses as possible.

### Eligibility Criteria

Only physicians practicing in Ontario and who have HIV patients in their care were included in this study. All participants provided informed consent.

### Data Collection

A total of 48 participants completed a cross-sectional survey hosted by FluidSurveys [14]. Questions were designed in consultation with HIV researchers, program evaluators, HIV educators, distance education specialists, community leaders living with HIV, and physicians with expertise in providing telemedicine services as well as HIV care.

The aims of the survey were to better understand current practices and perceived risks, benefits, and challenges in using telemedicine in HIV care. To do this, questions were organized into 5 sections: (1) general concepts of telemedicine, (2) perceived challenges to telemedicine use, (3) benefits of telemedicine, (4) perceived risks of telemedicine, and (5) information about the health care provider and their practice and patient population.

### Analysis

Data were analyzed using descriptive statistics and frequencies to examine the characteristics of invited participants and their responses to the survey items. Data are presented as total counts and percentages. For simplicity, physician perceptions were dichotomized by aggregating the categories “agree” and “strongly agree” to “agree” in the text, while “disagree” and “strongly disagree” were combined to “disagree.” In addition, “minor,” “moderate,” and “severe” barriers were consolidated to “barrier” in the text. Data synthesis and statistical analyses were performed using R 3.2.3 (R Core Team, Vienna Austria) [15].

### Ethics

This study was approved by the Research Ethics Board at St Michael’s Hospital in Toronto, Canada (REB#15-337). Per our study protocol, participation was completely voluntary. All questions were self-administered and anonymous. Participants were not compensated for completing the survey and were free to withdraw or not answer any questions they did not want to with no professional or other consequences.

## Results

**Recruitment and Participation**

Of the 51 physicians who were invited to take part in the Web-based survey, 50 (98%) consented to participate in the study. Two physicians who consented were removed because they did not answer any of the survey questions. All questions had 10% or fewer missing items, with most having only 4% missing.

### Demographic Characteristics of Respondents

Physician characteristics are presented in Table 1. About half of the invited physicians were generalists (44% family physician, 2% internal medicine, and 2% pediatrics), whereas 36% of physicians were specialists in infectious diseases and 9% were psychiatrists focusing in HIV. The majority of respondents primarily practiced in the Greater Toronto Area (69%) and represented a broad range of years in practice with 78% of physicians having at least ten years of experience. The respondents each represented a varying caseload of HIV patients.

### HIV Care Providers’ Use of Telemedicine

More than half of the physicians (62%, 29/47) reported that they used some form of telemedicine to care for HIV patients in their practice. Eighteen (38%, 18/47) respondents stated that they have never used telemedicine in their practice. Of the 29 respondents who were identified as having used telemedicine in their practice, telephone (86%, 25/29), email (69%, 20/29), and teleconsultation (24%, 7/29) were listed as frequent modalities used to care for patients. HIV care providers were more likely to use the telephone (69%, 20/29) to care for their patients as opposed to email (21%, 6/29) and teleconsultation systems (10%, 3/29; Table 1).

When requiring the assistance of a specialist in order to care for HIV patients, about half of the physicians (55%, 26/47) reported that they used telemedicine in order to consult with specialists. However, 21 respondents (46%, 21/47) stated that they have never used telemedicine to contact a specialist. The primary modalities used to consult with a specialist by the 26 respondents were email (58%, 15/26) and telephone (38%, 10/26).

**Table 1:** Characteristics of participants (N=48).

Participant characteristics		Number of respondents n (%)
**Location of practice, n=48**		
	Toronto or Ottawa	37 (77)
	Other locations in Ontario	11 (23)
**Type of physician, n=45**		
	Family medicine	20 (44)
	Infectious disease	16 (36)
	Other (ie, internal medicine, psychiatrist, etc)	9 (20)
**Years in practice, n=45**		
	<10	10 (22)
	10-15	8 (18)
	16-24	12 (27)
	≥25	15 (33)
**Percentage of patient case load that are people living with HIV^a^, n=45**		
	< 25%	14 (31)
	25-49%	12 (27)
	50-74%	9 (20)
	75-100%	10 (22)
**Modalities of HIV telemedicine currently used, n=29**		
	Phone consultations	25 (86)
	Email consultations	20 (69)
	Teleconsultation systems	7 (24)
	Other (ie, instant messaging software)	1 (3)
**Current use of telemedicine services, n=48**		
	Never or rarely	29 (60)
	Sometimes	11 (23)
	Often	7 (15)

^a^HIV: human immunodeficiency virus.

Twenty-six physicians (55%, 26/47) felt that there was a need to expand the use of HIV telemedicine services in Ontario; however, 20 physicians (43%, 20/47) were unsure of the need for an expansion of HIV telemedicine services, with 1 physician expressing a negative response to the development of telemedicine services in Ontario. Whereas only 26 physicians expressed interest in developing telemedicine services for HIV patients, many physicians (28/46, 61%) stated they served patients who could benefit from telemedicine. There were also a significant number of physicians who stated that they provided care to a number of patients who had difficulties traveling because of physical disabilities or mental health issues (82%, 37/45 and 72%, 34/47 respectively). Also, 74% (34/46) of those surveyed stated they had patients who traveled more than 100 kilometers for a visit.

### Physicians Perceived Challenges to Telemedicine Use in Their HIV Patients

Physicians endorsed various challenges relating to the use of telemedicine when caring for HIV patients (Table 2). Most notably, 83% (38/46) of physicians felt that they could not adequately assess the health of a patient via telemedicine. Sixty two percent (28/45 respondents) reported that telemedicine took too much time, and 60% (27/45 respondents) felt they lacked the technology to use telemedicine in their practice. The majority of respondents (76%, 34/45) believed that their patients did not have access to the necessary equipment needed to use telemedicine services. Many physicians cited other challenges such as confidentiality (60%, 27/45), lack of remuneration (62%, 28/45), concerns that patients will abuse telemedicine services (71%, 32/45), and medicolegal concerns (51%, 23/45). However, an absence of patients that would benefit from telemedicine was not identified as a barrier; 75% (33/44) of physicians stated that lack of need did not prevent them from using telemedicine.

### Perceived Risks of Using Telemedicine in Patients With HIV

Respondents expressed opinions regarding the perceived risks of using telemedicine for patients living with HIV (Table 3). Many respondents (65%, 28/43) agreed that patients may not “feel adequately connected” to them as a provider with the use of telemedicine. Fifty-eight percent (25/43 respondents) agreed that HIV patients would receive poorer quality assessments with the use of telemedicine. However, most physicians (77%, 33/43) disagreed with the statement that HIV patients would feel more social isolation with the use of telemedicine. Also, the majority of the respondents (67%, 29/43) disagreed with the statement that remote patients would lose the opportunity to come visit their practice.

### Perceived Benefits of Using Telemedicine to Care for HIV Patients

The majority of physicians stated that HIV patients would benefit from the use of telemedicine (Table 4). There was unanimous agreement among respondents (100%) when assessing telemedicine’s ability to reduce patients’ travel times. Eighty-three percent (38/46) of respondents agreed with the premise that using telemedicine can reduce a patients’ exposure to the stigma of having HIV. Additionally, 65% (30/46) of respondents agreed that the use of telemedicine can increase the patients’ privacy. Many physicians also agreed that both the quality of care (61%, 28/46) and efficacy of patient care (67%, 31/46) could improve in HIV patients through the use of telemedicine. Eighty-five percent (39/46) of respondents agreed with the statement that telemedicine will be able to increase the number of times patients are able to interact with their physician as well as agreed with its ability to improve access and timeliness to care.

**Table 2:** Participants’ perspectives on challenges to using telemedicine.

Perceived challenges to using telemedicine	No barrier n (%)	Minor barrier n (%)	Moderate barrier n (%)	Severe barrier n (%)
It takes too much time	17 (38)	20 (44)	6 (13)	2 (4)
I lack access to the necessary technology	18 (40)	9 (20)	15 (33)	3 (7)
I have no patients that require telemedicine services	33 (75)	7 (16)	1 (2)	3 (7)
Most of my patients do not have access to the necessary equipment for telemedicine	11 (24)	12 (27)	19 (42)	3 (7)
I have concerns about confidentiality	18 (40)	17 (38)	7 (16)	3 (7)
There is too much diversity in my practice to adopt telemedicine as a regular practice	26 (58)	13 (29)	5 (11)	1 (2)
I cannot adequately assess a patient using telemedicine	8 (17)	19 (41)	15 (33)	4 (9)
I have concerns about remuneration for telemedicine	17 (38)	12 (27)	10 (22)	6 (13)
I worry that patients will abuse the use of telemedicine to communicate with me	13 (29)	16 (36)	15 (33)	1 (2)
I have medicolegal and licensing concerns about using telemedicine	22 (49)	15 (33)	8 (18)	0

**Table 3:** Participants’ perspectives on risks of telemedicine.

Perceived risks of using telemedicine	Strongly disagree n (%)	Disagree n (%)	Agree n (%)	Strongly agree n (%)
I am concerned that telemedicine will increase the social isolation experienced by people living with HIV^a^	4 (9)	29 (67)	7 (16)	3 (7)
Patients may not feel adequately connected to me as a health care provider	3 (7)	12 (28)	22 (51)	6 (14)
Patients will receive lesser quality assessments	3 (7)	15 (35)	20 (47)	5 (12)
Patients may not understand my instructions	3 (7)	20 (47)	19 (44)	1 (2)
Remote patients will lose the opportunity to come see me in person if they prefer it	3 (7)	26 (60)	13 (30)	1 (2)

^a^HIV: human immunodeficiency virus.

**Table 4:** Participants’ perspectives on benefits of telemedicine.

Perceived benefits of using telemedicine	Strongly disagree n (%)	Disagree n (%)	Agree n (%)	Strongly agree n (%)
Reduces their exposure to stigma (in rural areas, for example, acquaintances or family would not see them visit medical services)	0	8 (17)	34 (74)	4 (9)
Increases their privacy	0	16 (35)	26 (57)	4 (9)
Reduces travel time	0	0	15 (33)	31 (67)
Improves quality of patient care	1 (2)	17 (37)	21 (46)	7 (15)
Improves efficacy of patient care	0	15 (33)	22 (48)	9 (20)
Increases the number of times we can interact	0	7 (15)	29 (63)	10 (22)
Improves access and timeliness to care	0	7 (15)	28 (61)	11 (24)

## Discussion

### Principal Findings

The aim of this study was to help describe physicians’ perceptions of the use of telemedicine to care for patients living with HIV and the perception they have of their patients’ use of telemedicine; the barriers and facilitators. In terms of benefits, physicians agreed across the board that telemedicine services would decrease travel times and could likely reduce patient experience of HIV-related stigma at appointments. Physicians also felt that telemedicine would increase access and efficiency of care, while benefiting patients who have difficulty travelling due to physical and mental impairments.

We discovered some consistent trends when evaluating the perceived challenges physicians reported to using telemedicine in their practice. Physicians most notably reported apparent issues around lack of time for telemedicine, lack of necessary technology for the patient and the provider, as well as concerns about confidentiality, remuneration, and inability to adequately assess patients using the service. These results echo some of the current sentiments regarding the uptake of telemedicine into general medical practice [1-5]. Physicians were also concerned about patients not feeling adequately connected to them as a health care provider when using telemedicine, and to a lesser extent, believed that patients would receive lesser quality assessments.

Although there was consistent agreement around possible benefits of telemedicine for HIV care, our findings highlight many perceived challenges and risks that must be addressed before HIV telemedicine is likely to expand dramatically in practice. Confidentiality, privacy, and remuneration were reported as key challenges to physicians adopting telemedicine in Ontario. These challenges may primarily be due to a lack of information on the physicians’ part in regards to the telemedicine services at their disposal and have less to do with regulation limitations, or limitations of the technology. Previous research has shown that physicians are less likely to use telemedicine services on a regular basis if they are not adequately compensated for their time and effort [13,16]. Addressing these perceived barriers to the implementation of telemedicine services is a complex problem that requires assistance from many sources including health care institutions, policy makers, physicians, and patients alike.

Some of these data reflect current themes in distance education; the impact of in-person consultation is glamorized, whereas “Web-based presence” is misunderstood as impersonal. In many responses, it seems that physicians fear that the introduction of telemedicine into practice will be a replacement to in-person consultations and not a compliment to current practices. The nature of HIV as a complex syndrome of various clinical manifestations may also be contributing to the physicians’ hesitation to endorse telemedicine. For example, people living with HIV may have complex social, financial, and psychological concerns that seem less amenable to telemedicine techniques when a provider is inexperienced with these remote modalities.

In Canada, the Canada Health Infoway, which is a national initiative, was created to expedite the development and adoption of telemedicine services while addressing reported barriers to the implementation of eHealth systems. Through the use of provincial partners like the Ontario Telemedicine Network, the infrastructure is in place to provide telemedicine services to those receiving care for HIV. The use of telemedicine to treat other chief health complaints has shown positive results as evidenced by the success of Telehomecare, Telestroke, and Teledermatology programs in Ontario [17].

Telemedicine networks in Canada offer education for physicians and their staff around remuneration, the technology of telemedicine, as well as ways to incorporate telemedicine alongside in-person care. Physicians and health care providers may need ongoing training and support in the form of distance educational sessions to gain up-to-date and meaningful instruction on the benefit of telemedicine services and how to seamlessly integrate them into their practices. By providing physicians with evidence-based research on the growing need for innovative care and the benefits of implementing telemedicine services, their perspectives may change and allow for a greater adoption of the service in HIV care provision.

### Limitations

In terms of collecting respondent characteristics, we collected few demographic identifiers in order to protect confidentiality of northern providers that may be using telemedicine more often or more proficiently than tertiary providers. We also did not gather the ages or genders of physicians who responded in our survey, which could possibly be a predictor of telemedicine perceptions. We also were not able to gather opinions of all physicians practicing HIV care in Ontario, as there is not a full, up-to-date registry of these physicians.

### Conclusions

From the perceptions of physicians, telemedicine shows promise in the care of patients living with HIV. More than half of the respondents are already using telemedicine. Whereas many physicians are concerned about their ability to fully assess the health of a patient via telemedicine, most physicians do see a need for it to reduce patient travel times, reduce exposure to stigma, and improve efficiency and timely access to care. Challenges and risks such as technological gaps, confidentiality, and medicolegal concerns must be addressed for physicians to feel more comfortable using telemedicine. Further research is warranted to determine the levels and needs for training of physicians and patients on various telemedicine modalities and technologies. Also, there is a need to compare and contrast the data collected with research evidence in telemedicine uptake in other health areas such as wound care, diabetes, and counseling.

## Abbreviations

HIV: human immunodeficiency virus
